# Myocardial extracellular volume fraction quantified by cardiovascular magnetic resonance is increased in hypertension and associated with left ventricular remodelling

**DOI:** 10.1186/1532-429X-18-S1-P282

**Published:** 2016-01-27

**Authors:** Minjie Lu, Yan Zhang, Jing An, Shihua Zhao, Peter Kellman

**Affiliations:** 1 grid.415105.4Magnetic Resonance Imaging, Fuwai Hospital, Beijing, China; 2 Siemens Shenzhen Magnetic Resonance Ltd, Shenzhen, China; 3 Cardiovascular and Pulmonary Branch, National Heart, Lung and Blood Institute, National Institutes of Health, US Department of Health and Human Services, Bethesda, MD USA

## Background

There are limited data reported on the myocardial ECV in an essential hypertension patient population. The purpose of this study was to determine whether extracellular volume fraction (ECV) quantification by cardiac magnetic resonance (CMR) can demonstrate left ventricle (LV) abnormalities in a cohort of consecutive hypertension (HTN) patients, and to investigate the relationship between ECV and global LV function and LV remodelling.

## Methods

ECV quantification with cardiac MR imaging was prospectively performed in 134 consecutive HTN patients and 97 healthy subjects. The HTN patients were separated into two subgroups according to LGE present or not. Individual and regional ECV were compared to the regions on LGE images using a 17-segment model. Statistical comparisons of LV global functional parameters, and ECV were carried out using the Pearson correlation, Student *t* test, and multiple regressions.

## Results

In HTN group, 70.1 %( 94/134) was LGE negative and 29.9% (40/134) was LGE positive. The average ECV in healthy controls and LGE negative patients were 26.9 ± 2.67 % and 28.5 ± 2.9% (*P*<0.001), respectively. The differences of ECV reached statistical significance among the regions of LGE area, LGE-Peri area, LGE remote area and the normal area of the control group(all *P*<0.05) in LGE positive subgroup. Global ECV significantly correlated with LVEF (r=-0.466, *P* < 0 .001) and LV hypertrophy (r= 0.667, *P* < 0.001).

## Conclusions

ECV imaging by cardiac MR imaging can identify LV abnormalities at an early stage in HTN patients without LGE. These abnormalities may reflect increase in diffuse myocardial fibrosis and are associated with LV remodelling.

